# Single-photon emission computed tomography/computed tomography imaging of RAGE in smoking-induced lung injury

**DOI:** 10.1186/s12931-019-1064-4

**Published:** 2019-06-10

**Authors:** Monica P. Goldklang, Yared Tekabe, Tina Zelonina, Jordis Trischler, Rui Xiao, Kyle Stearns, Krissy Rodriguez, Alexander Shields, Alexander Romanov, Jeanine M. D’Armiento, Lynne L. Johnson

**Affiliations:** 1 Department of Anesthesiology, Columbia University, New York, NY USA; 2 Department of Medicine, Columbia University, New York, NY USA; 3 Department of Physiology and Cellular Biophysics, Columbia University, New York, NY USA; 4 Institute for Comparative Medicine, Columbia University, New York, NY USA

**Keywords:** Emphysema, Cigarette smoke, Rabbit, Damage-associated molecular pattern, Imaging, Receptor for advanced glycation end products, HGMB1

## Abstract

**Background:**

Expression of the Receptor for Advanced Glycation Endproducts (RAGE) initiates pro-inflammatory pathways resulting in lung destruction. We hypothesized that RAGE directed imaging demonstrates increased lung uptake in smoke-exposure.

**Methods:**

After exposure to room air or to cigarette smoke for 4-weeks or 16-weeks, rabbits were injected with ^99m^Tc-anti-RAGE F(ab’)_2_ and underwent Single-Photon Emission Computed Tomography/Computed Tomography (SPECT/CT) imaging. Lung radiotracer uptake was calculated as percent injected dose (%ID). Lungs were dissected for gamma well counting and histological analysis.

**Results:**

^99m^Tc-anti-RAGE F(ab’)_2_ SPECT/CT imaging demonstrated increased lung expression of RAGE with smoke exposure compared to room air control at 4-weeks: Room air right (R) 0.75 ± 0.38%ID, left (L) 0.62 ± 0.32%ID vs. Smoke exposed R 0.17 ± 0.03, L 0.17 ± 0.02%ID (*p* = 0.02 and 0.028, respectively). By 16-weeks of smoke exposure, the uptake decreased to 0.19 ± 0.05%ID R and 0.17 ± 0.05%ID L, significantly lower than 4-week imaging (*p* = 0.0076 and 0.0129 respectively). Staining for RAGE confirmed SPECT results, with the RAGE ligand HMGB1 upregulated in the macrophages of 4-week smoke-exposed rabbits.

**Conclusions:**

RAGE-directed imaging identified pulmonary RAGE expression acutely in vivo in an animal model of emphysema early after smoke exposure, with diminution over time. These studies document the extent and time course of RAGE expression under smoke exposure conditions and could be utilized for disease monitoring and examining response to future RAGE-targeted therapies.

**Electronic supplementary material:**

The online version of this article (10.1186/s12931-019-1064-4) contains supplementary material, which is available to authorized users.

## Background

Inflammation seen in smoking related lung disease is triggered in part by receptor for advanced glycation end-products (RAGE) [1] a multiligand receptor that is expressed by parenchymal lung cells, vascular cells, and inflammatory cells and binds to oxidized proteins and lipids, as well as the ligands high mobility group box 1 (HMGB1), S100A8, S100A9, and S100A12 in response to smoke and pollutant exposure [2–15]. RAGE ligand binding activates intracellular pathways leading to the release of cytokines linked to inflammation and cell death [7, 12, 15]. RAGE expression is up-regulated in the alveolar and bronchial epithelium [8, 16] and pulmonary artery smooth muscle and pulmonary artery endothelium [13] of mice exposed to cigarette smoke. Increased RAGE staining in the alveolar walls of patients with chronic obstructive pulmonary disease (COPD) has been demonstrated to inversely correlate with lung function [4]; increased levels of the RAGE ligand HMGB1 in the bronchoalveolar lavage (BAL) fluid from smokers with COPD also has a similar correlation with worsening lung function [3].

Chest computerized tomography (CT) is the currently available imaging platform to assess lung disease after destruction has occurred. Utilizing tissue density, CT can identify structural damage such as airway enlargement and airway wall thickening and remodelling that occurs in emphysema. Aiming to develop an imaging approach that provides information on both anatomy and cellular processes related to disease pathogenesis and evolution would add useful clinical information in the evaluation of the disease course, prognosis assessment, and response to treatment. Hybrid imaging systems combining single photon emission computed tomography (SPECT) with CT or positron emission tomography (PET) with CT provide platforms to localize and quantify uptake of radiolabelled probes targeting biological markers important in lung disease.

We recently described the development and detailed characterization of a rabbit model of cigarette smoke induced COPD with many of the histopathological and lung functional characteristics of human disease including a neutrophilic airway infiltrate, increased pulmonary compliance, and airspace enlargement [17]. In this model, we also described the in vivo time course of the upregulation of the apoptosis-associated molecule Annexin V, demonstrating a sustained increase in smoke-exposed rabbits after 4-weeks and 16-weeks of smoke exposure when quantified by SPECT/CT, correlating with cellular apoptosis on ex vivo tissue analysis. In a set of parallel experiments, we now report on the time course of RAGE expression in this animal model of COPD. We developed an anti-RAGE antibody targeting a unique site on the extracellular V domain of the RAGE receptor. When radiolabelled with technetium-99 m (^99m^Tc), we have shown the sensitivity and specificity to detect the RAGE signal on in vivo SPECT/CT imaging in diseases associated with increased RAGE expression including atherosclerosis, diabetic peripheral vascular disease, and myocardial reperfusion injury [18–23]. We hypothesized that RAGE directed SPECT/CT imaging in these rabbits may be a useful tool to identify the extent, magnitude, and time course of RAGE expression using this relevant animal model of emphysema.

## Methods

### Animals

All animal experiments were performed with the approval of the Institutional Animal Care and Use Committee of Columbia University (Columbia University IACUC AC-AAAP3404). Mature female New Zealand White Rabbits (approximately 6 months of age with a weight 1.3–1.8 kg) were obtained from Harlan Laboratories (Indianapolis, IN, USA) and Charles River Laboratories (Wilmington, MA, USA). These imaging studies were performed in parallel with the extensive characterization of the rabbit smoke exposure model presented within our Annexin V targeted SPECT/CT imaging studies [17].

### Exposure of rabbits to cigarette smoke

Following acclimatization for 48 h in the animal facility, rabbits underwent smoke exposure utilizing the TE-10 Teague Smoking Apparatus (Teague Enterprises, Woodland, CA, USA) with University of Kentucky 3R4F Reference Cigarettes (University of Kentucky, Lexington, KY, USA). Total particulate matter was maintained at 100–150 mg/m^3^ as measured by gravimetric analysis. Rabbits underwent smoke exposure 4 h per day, 5 days per week for 4 or 16-weeks including the day prior to imaging and/or sacrifice (4-weeks *n* = 5, 16-weeks *n* = 4) [17]. Age and weight matched rabbits breathing room air were followed over the same time intervals as controls (4-weeks *n* = 4, 16-weeks *n* = 4). The development of emphysema, including lung structural, mechanical, and inflammatory changes, in these rabbits has been reported [17]. Animals underwent imaging with both RAGE and apoptosis targeting imaging agents, with the exception of two animals in the 4-week room air group, and one animal in each of the 4-week smoke-exposure, 16-week room air, and 16-week smoke exposure groups.

### Preparation of radiotracer

The peptide sequence and production of the murine hybridoma has been established [18]. Direct coupling of diethylenetriamine pentaacetic acid (DTPA) (bicyclic anhydride) to anti-RAGE F(ab′)_2_ antibody fragments for ^99m^Tc labelling was performed as previously described [18, 24]. For details see Additional file 1.

### Blood pool clearance and biodistribution

Ear vein catheters were placed in both ears of 2 normal rabbits. Into one ear vein, an average dose of 3.5 mCi (129.5 MBq) of ^99m^Tc anti-RAGE F(ab’)_2_ was injected, and from the opposite ear vein blood samples were withdrawn at 2, 5, 10, 15, 20, 30, 45, 60, 90, 120, 180, 240, and 300 min. From each tube, 50 μl aliquots were pipetted into pre-weighed tubes and counted in a gamma well counter. The counts vs. time were averaged for each time point and plotted. Rabbits were sacrificed and organs removed and weighed and samples counted in the gamma well counter.

### Radiotracer injection and imaging

Rabbits were injected with 3.47 ± 0.29 mCi (128.4 ± 10.73 MBq) ^99m^Tc anti-RAGE F(ab’)_2_ via an ear vein catheter and approximately 6 h later SPECT imaging was performed on the Bioscan (Mediso) nanoSPECT fitted with 2 LEUHR parallel hole collimators for 35 min per scan. For imaging, rabbits were sedated with ketamine 35 mg/kg (Henry Schein Animal Health, Dublin, Ohio, USA) and xylazine 2.5 mg/kg (Lloyd, Inc., Shenandoah, Iowa, USA) in a single intramuscular injection. Within 3 days of the SPECT scan, rabbits underwent a spiral chest CT on a Siemens 64 slice Biograph scanner. For details see Additional file 1.

### Tissue processing

At the completion of the final scan, rabbits were sacrificed with a fatal dose of pentobarbital and phenytoin at a dose of 100 mg/kg pentobarbital (Euthasol, 390 mg pentobarbital sodium and 30 mg phenytoin sodium per mL, Virbac AH, Inc., Fort Worth, Texas, USA). The trachea was cannulated and the lungs pressure perfused with 10% formalin to 25 cm H_2_O. Tissue from each lung region (right upper, right lower, left upper and left lower) was formalin fixed and paraffin embedded for sectioning and staining. Additional sections from both lungs were dissected and weighed, and the radioactivity determined in a gamma well counter (Wallac Wizard 1470, PerkinElmer, Waltham, MA, USA) and expressed as the percentage of injected dose per gram (%ID/g) tissue. Radiotracer activity in the samples was corrected for background, decay time, and tissue weight. To reduce effect of variability of values and sampling errors, samples from both lungs for each animal were pooled and the results averaged.

### Image processing

See Additional file 1.

### RAGE immunohistochemistry

Quantitative immunohistochemical analyses of the lung sections were performed. Sections were deparaffinized in xylene, treated with 0.3% hydrogen peroxide for 20 min, and incubated in protein-free block (Dako Inc., Carpinteria, CA, USA) for 10 min to inhibit the non-specific binding of primary antibody. Staining for RAGE was performed using goat anti-RAGE polyclonal antibody (ab7764, Abcam, Cambridge, MA, USA) at a dilution of 1:250 in BSA, with a low pH (6.0) sodium citrate buffer for antigen retrieval and a one hour incubation in a 37 °C water bath. Secondary staining was performed with HRP-conjugated anti-goat secondary antibody, followed by diaminobenzidine (DAB substrate kit for peroxidase; Vector Laboratories, Burlingame, CA, USA), and counterstaining with Gill’s hematoxylin solution. Immunohistochemical analyses of the lung sections were performed using a Nikon Eclipse 50i upright microscope (Tokyo, Japan). Photographs were opened into Image-Pro Plus software (Media Cybernetics Inc., Silver Spring, MD, USA). The brown staining from the HRP coated secondary anti-RAGE antibody was quantified based on color recognition (brown) for entire fields (10X) on three sections. The percentage RAGE staining was determined as area of brown staining per high powered field and results from the 3 serial sections averaged.

### Immunofluorescence

HMGB1 immunofluorescence staining was performed on lung sections using mouse monoclonal anti-HMGB1 antibody (Anti-HMGB1 antibody ab184532, Abcam). Sections were blocked for 1 h at room temperature with 5% Normal Donkey Serum. Primary antibody was diluted to 1:400 in 5% Normal Donkey Serum, and incubated overnight at 4 °C. Secondary antibody was incubated for 1 h at room temperature (AlexaFluor 488 donkey anti-mouse IgG, 1:1000, Thermo Fisher Scientific). Hoechst staining was performed for 10 min, and slides washed and imaged with a Nikon Eclipse Ti inverted microscope. HMGB1 was qualitatively assessed with regards to the cell of localization.

### Statistical analysis

Uptake of ^99m^Tc anti-RAGE F(ab’)_2_ for each lung was averaged for each group. Comparisons were made between the 4-week room air and smoke exposed groups, 16-week room air and smoke exposed and between the 4 and 16-week smoke exposed groups using one-way analysis of variance (ANOVA) with Tukey’s multiple comparison test. Comparisons were deemed statistically significant if *p* < 0.05. Values for tracer uptake as %ID (from scan) for each lung and RAGE IHC quantification were plotted vs %ID/g for each lung (average of samples) using Pearson’s Correlation. Prism 7 for Mac OS X was utilized for all statistical analysis.

## Results

### Blood pool clearance and biodistribution

**Fig. 1:**
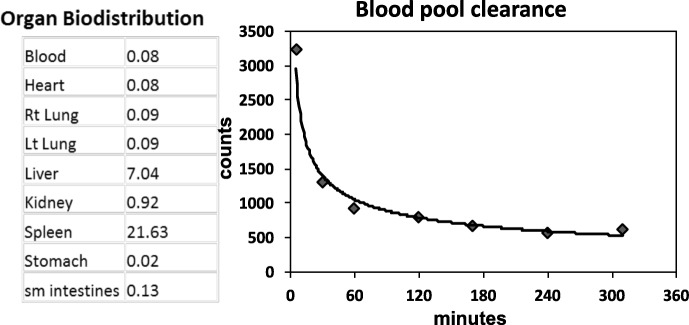
Organ biodistribution of ^99m^Tc anti-RAGE F(ab’)_2_. Organ biodistribution is displayed as %ID/g tissue in a room air exposed rabbit. Blood pool clearance curve is provided on the right, with each point representing the average values from 2 room air exposed rabbits

Two normal rabbits were utilized for blood pool and organ biodistribution analyses. Blood pool clearance curves were bi-exponential and the calculated biological half life (t½) for the first component = 23 min and for the second component = 250 min (Fig. 1). These results were similar to reported values in mice and pigs [18, 23] and based on these results the optimal time for imaging was selected as 6 h after injection. The organ with the highest uptake of ^99m^Tc anti-RAGE F(ab’)_2_ was the spleen followed by the liver. The remaining organs including the lungs, blood, and kidneys all had less than 1% ID/g tissue (Fig. 1).

### Lung uptake of ^99m^Tc anti-RAGE F(ab’)_2_ on SPECT/CT scans

**Fig. 2:**
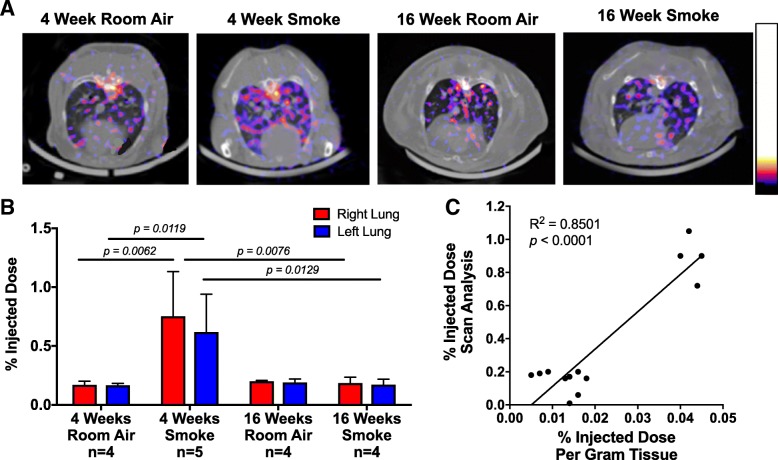
Increased RAGE signal on SPECT/CT in 4-week smoke-exposed rabbits. **a** Transverse SPECT/CT slices from the mid lung fields for 4-week and 16-week smoke-exposed and room air control rabbits. The tracer uptake is highest in the lungs of 4-week smoke exposed rabbits. The color table scaling reference bar for the SPECT images shows the upper and lower threshold levels selected to optimally display lung uptake of radiotracer. The (**b**) %ID for the right and left lung quantifications, data provided as mean ± SD, *n* = 4–5 per group. **c** Gamma well counting significantly correlates with the %ID as calculated on imaging analysis across all imaging situations

Rabbits were exposed to cigarette smoke and fully characterized with regards to lung inflammation and emphysema development [17]. Transverse slices from the ^99m^Tc anti-RAGE F(ab’)_2_ SPECT/CT scans taken in the mid lung fields are shown in Fig. 2a. The bar graphs in Fig. 2b show the data expressed as mean ± SD for the sums of activity for the serial transverse sections for each lung. The uptake as %ID for each lung was higher in the 4-week smoke exposed rabbits: (0.75 ± 0.38%ID R lung, 0.62 ± 0.32%ID L lung) compared to the 4-week room air exposed rabbits (0.017 ± 0.03%ID R lung, 0.017 ± 0.02%ID L lung) (*P* = 0.02 and 0.028 for right and left comparisons, respectively). The lung uptake at 16-weeks in the smoke exposed rabbits was 0.19 ± 0.05 for the R lung and 0.17 ± 0.05 for the L lung. These values were not different from 16-week room air exposed animals and significantly lower than 4-week smoke exposure (P = 0.02 vs. R lung 4-week smoke exposed and *P* = 0.03 vs. L lung 4-week smoke exposed). When values (*n* = 13) for tracer uptake in right and left lung for 2–3 experiments per condition (room air 4- and 16-weeks, smoke exposure 4- and 16-weeks) were plotted vs. average of lung sections gamma well counted for each experiment, there was a significant correlation (*R*^2^ = 0.8501, *P* < 0.0001, Fig. 2c).

### Lung volumes and Hounsfield units

In the rabbit, it is well established that R lung volumes are larger than the L lung due to the area occupied by the heart in the L thorax [25]. There were no significant differences between the lung volumes for the 4-week room air and 4-week smoke exposed rabbits for either the R lung: 16.42 ± 1.54 cc vs. 15.96 ± 2.23 cc, or the L lung: 13.12 ± 2.85 cc vs. 15.15 ± 2.46 cc. The 16-week room air rabbit lung volumes were not significantly different from the 4-week room air exposed rabbits: 17.55 ± 1.28 for the R lung and 14.46 ± 1.91 for the L lung. In contrast, the volumes for the 16-week smoke exposed rabbits were significantly larger than for the 16-week room air exposed rabbits: 26.32 ± 5.54 cc for the R lung (*P* = 0.01 vs. room air exposed) and 20.86 ± 5.80 for the L lung (*P* = 0.05 vs. room air exposed). Lung volumes of the 16-week smoke exposed rabbit were significantly larger than the 4-week smoke exposed rabbit (*P* = 0.01 R lung, *P* = 0.05 L lung). While the lung volumes were larger in the 16-week smoke exposed rabbits compared to the room air exposed rabbits, the mean values for lung density measured in Hounsfield units were not significantly different. Hounsfield values for the R lung for the room air vs. smoke exposed rabbits were − 516 ± 112 vs. -601 ± 57 (*P* = 0.29) and for the L lung − 564 ± 73 vs. -631 ± 86 (*P* = 0.32). In addition, there was no significant difference in Hounsfield values comparing 4-week smoke exposed (4-week R lung − 539 ± 41, L lung − 586 ± 48) and 16-week smoke exposed rabbits (R lung *P* = 0.19, L lung *P* = 0.47).

### Immunohistochemistry

**Fig. 3:**
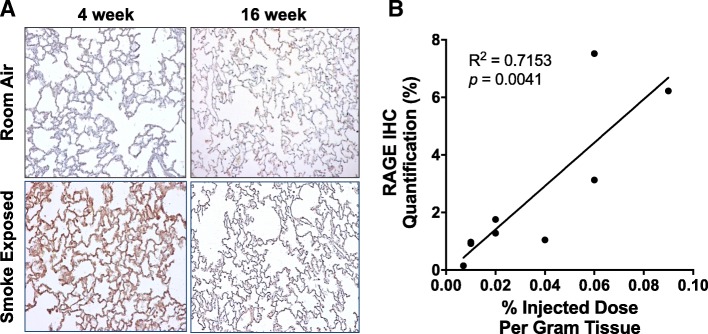
Increased RAGE staining by immunohistochemistry in the 4-week smoke-exposed rabbit lung. **a** Increased RAGE staining (brown colour) in the alveolar epithelium and inflammatory cells of 4-week smoke-exposed rabbits. There is low uptake in the 16-week smoke-exposure lung tissue as well as in the room air control rabbits. **b** IHC quantification, represented as % brown staining per high powered microscopic field shows statistically significant correlation between RAGE staining and RAGE radiotracer uptake per gram of tissue. Images taken at 10X magnification

**Fig. 4:**
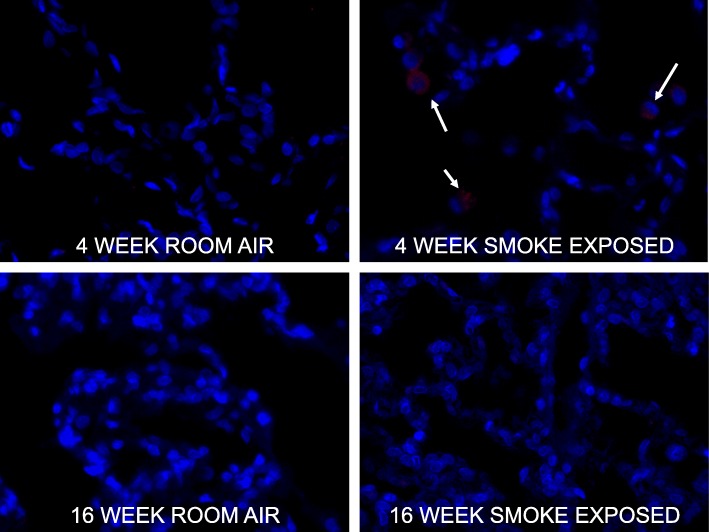
Increased HMGB1 immunofluorescence in alveolar macrophages after 4-weeks of smoke exposure. In 4-week smoke exposed rabbit lung, HMGB1 (red colour) localizes to the macrophage cytoplasm. Nuclei are stained blue. Representative images are provided, with white arrows denoting areas of cells with increased HMGB1 staining. Images were taken at 20X magnification

Quantitative immunohistochemistry staining was performed for RAGE on lung tissue after the rabbits were sacrificed. Alveolar epithelial cells exhibit strong positive staining for RAGE (Fig. 3). The highest % staining was on the lung samples from rabbits exposed to cigarette smoke for 4-weeks. There was low staining on the lung tissue from rabbits exposed to room air for 4-weeks. In addition, the higher levels of lung tissue RAGE staining fell by about half from 4-weeks to 16-weeks of continuous smoke exposure. These results correlate well with the scan data, with a statistically significant correlation between RAGE gamma well counts and RAGE immunohistochemistry. On further examination, the RAGE ligand HMGB1 localized to the macrophage cytoplasm upon 4-weeks of smoke exposure (Fig. 4), but does not localize to the cytoplasm under room air or 16-week smoke exposure conditions. RAGE staining correlates with the imaging signal observed, and an increased level of the RAGE ligand HMGB1 was detected in macrophages following early smoke exposure.

## Discussion

In a rabbit model of smoke induced lung injury with similarities to the human disease [17], we demonstrated increased lung uptake of ^99m^Tc anti-RAGE antibody on SPECT/CT scans after 4-weeks of smoke exposure compared to control room air exposed rabbits. After 16-weeks of smoke exposure, RAGE antibody uptake had fallen back to room air control levels. Ex vivo analysis of RAGE correlated with the imaging findings. Interestingly, RAGE uptake increases prior to inflammation [17], suggesting that RAGE upregulation is an early event in disease pathogenesis. In chronic smoke exposure conditions, RAGE antibody uptake decreases, but a macrophage and neutrophilic inflammation persists [17]. Therefore, this imaging tool likely reflects early signaling processes in smoke-induced lung injury that precede the development of lung destruction.

There were no significant differences in lung volumes between the room air and smoke exposed rabbits at 4 weeks while the lung volumes were higher in the 16 week smoke exposed rabbits compared to 4 week smoke exposed [17]. This difference most likely represents early development of COPD in these rabbits. Uptake of the radiotracer was consistently higher in the right lung compared to the left lung, explained by the anatomically determined higher volume of the right compared to the left lung. However, it should be noted that lung tissue densities as Hounsfield units were not significantly lower in the lungs of the 16 week smoke exposed rabbits and therefore we do not think that the lower uptake of the RAGE targeting tracer after 16-weeks of smoke exposure can be explained solely by the loss of parenchymal tissue. These results provide important information on the time course of pulmonary RAGE expression in smoke exposure lung disease, and the possible window for therapy to block this inflammatory pathway and prevent tissue damage.

Our rabbit model of smoke exposure exhibited increased HMGB1 staining in alveolar macrophages after 4-weeks of smoke exposure, consistent with the chronic murine smoke exposure model [26]. In the acute (3 days) murine smoke exposure model, HMGB1 is upregulated in pulmonary epithelial cells and HGMB1 translocates from the nucleus to the cytoplasm for later extracellular release and ligand engagement with DAMPs [27]. HMGB1 activates NF-κB and JNK/p38 MAP kinase pathways through a TLR4/MyD88 dependent pathway [27]. The activation of TLR4 is a critical upstream signalling event in the smoke-induced induction of the collagenase MMP-1 in emphysema development [28].

Clinicians diagnose and classify chronic lung disease with pulmonary function tests and gas exchange (FEV1, lung volumes, diffusing capacity) and with CT scans. The latter can identify pulmonary structural changes such as emphysematous blebs or airway enlargement, fibrosis and air trapping after damage has occurred. This current paper reports radionuclide imaging targeting molecular pathways involved in the initiation and progression of inflammatory lung disease. Based upon the data provided above, induction of RAGE is an early pro-inflammatory change that does not persist with long-term smoke exposure. This is as opposed to the persistent upregulation of apoptosis through the duration of smoke exposure demonstrated with the molecular imaging of Annexin V in the rabbit smoke exposure model system [26]. It suggests that there are temporal changes in the early and late inflammatory responses in the chronic smoke exposure model, and that the change in signal is not solely due to an increased inflammatory response or cellular recruitment into the lung as a consequence of smoke exposure. To our knowledge this is the first study to examine in an animal model the time course for RAGE expression in chronic smoke exposure conditions. It also establishes that this imaging technology is capable of following changes in inflammatory and destructive processes over time.

Damage-associated molecular patterns (DAMPs) are endogenous molecules released by lung tissue in response to inhalation of toxic substances and activate the local native immune responses to express pattern-recognition receptors (PAMP) that bind the DAMP ligands [7]. Receptor/ligand binding initiates downstream pathways that amplify proinflammatory and apoptotic responses [7, 12]. The RAGE transmembrane receptor is one of these PAMPs and binds multiple molecules including HMGB1, and S100A12 [3, 12, 14]. The receptor/ligand binding not only stimulates downstream pathways but also leads to sustained local production of ligands and further upregulation of RAGE expression. This response is innate and exists to protect lung integrity. While the binding of RAGE ligands can initiate a vicious cycle of more damage, increased release of DAMPS, and greater inflammation through activation of macrophages and recruitment of neutrophils, the role of RAGE in continued inflammation and lung damage is an open question. Although the inhalation of toxins can initiate an inflammatory response, additional genetic factors may also be involved in disease progression and severity [8]. In this rabbit model our imaging and histology data suggest that after the initial response of increased RAGE expression, chronic cigarette smoke exposure does not perpetuate RAGE expression.

Many organs of the body and in particular the vascular endothelium of major arteries normally express RAGE in low levels [29, 30]. Constitutive expression of RAGE appears to be higher in lungs than other body organs in many species including humans [5, 8, 31]. These findings suggest that RAGE plays a complex role in activation and/or suppression of the inflammatory response in lung disease. In normal human lung tissue RAGE immunoreactivity is found in bronchiolar epithelia, type II alveolar pneumocytes, and alveolar macrophages as well as the endothelium of larger arteries [5]. Immunohistochemical staining of lung tissue from biopsies or lobectomies displayed increased AGE and RAGE staining in the alveolar walls of patients with smoke related pulmonary diseases [4]. Quantitative staining for RAGE on lung tissue samples correlated inversely with FEV1 [4]. Increased levels of the RAGE ligand HMGB1 in the bronchoalveolar lavage (BAL) fluid from smokers with COPD exhibited a similar correlation with lung function [3].

## Limitations

The current study uses a rabbit model and may be limited in application to humans. While the smoke induced changes found in the rabbit fairly closely mimic human disease and are closer than the mouse [17], the biodistribution of RAGE expression based on organ uptake of the radiolabelled antibody is different from what has been reported in humans especially the low level of constitutive expression in the lungs. The role of RAGE in development and progression of COPD as inducer vs. preventer of injury is complex and interplay of these may vary among species.

## Conclusions

In this rabbit model of cigarette smoke induced lung disease, RAGE expression increased early in smoke exposure but by 16-weeks was down to levels found in the room air exposed rabbits. This suggests that RAGE is involved in the early inflammation but not in sustained cigarette-smoke induced injury and appears to agree with a biomarker study showing low levels of sRAGE in lungs from patients with more advanced disease [32]. It has been hypothesized that the inflammatory process begun by RAGE signalling early in toxin exposure induces or activates other pathways including apoptosis and metalloproteinase expression and that these other activated processes lead to lung parenchymal destruction [12, 14, 15, 28]. The results described in the present study demonstrate the value of further development of molecular lung imaging to target biological processes important in the initiation and progression of COPD in human subjects. These findings exhibiting RAGE expression early on in injury are found in a relevant animal model of COPD [17] and suggest that potentially blocking this early initiation of RAGE in susceptible patients might be efficacious in reducing lung damage.

## Additional file

Additional file 1: Methods for Supplement. (PDF 31 kb)

## Abbreviations

%ID: Percent injected dose
^99m^Tc: Technitium
BAL: Bronchoalveolar lavage
COPD: Chronic obstructive pulmonary disease
DAMPs: Damage associated molecular patterns
DTPA: Diethylenetriamine Pentaacetic Acid
FEV1: Forced expiratory volume in one second
HMGB1: High mobility group box 1
IACUC: Institutional Animal Care and Use Committee
JNK kinase: c-Jun N-terminal kinase
MAP kinase: Mitogen-activated protein kinase
MMP: Matrix metalloproteinase
NF-κB: Nuclear factor kappa-light-chain-enhancer of activated B cells
PAMPs: Pathogen associated molecular patterns
PET/CT: Positron emission tomography/computed tomography
RAGE: Receptor for advanced glycation end products
SPECT/CT: Single-photon emission computed tomography/computed tomography
TLR4: Toll Like Receptor 4
