# Supplementary material for: Single-photon emission computed tomography/computed tomography imaging of RAGE in smoking-induced lung injury
Source: Respir Res. 2019 Jun 10;20:116. doi: 10.1186/s12931-019-1064-4 (PMC6558785; doi:10.1186/s12931-019-1064-4)
Supplement: Supplementary file 1 — Methods for Supplement. (PDF 31 kb) [file 12931_2019_1064_MOESM1_ESM.pdf]

Methods for Supplement

Preparation of radiotracer: The peptide sequence and production of the murine hybridoma has been established [16]. Direct coupling of diethylenetriaminepentaacetic acid (DTPA) (bicyclic anhydride) to anti-RAGE $F(ab')_2$ antibody fragments for radiolabelling with ^{99m}Tc was performed as following [16, 23]. An aliquot of modified anti-RAGE $F(ab')_2$ (1 to 2 mg) was reacted with 5-fold molar excess of bicyclic anhydride of DTPA in 0.5 mL dimethyl sulfoxide for 30 minutes while stirring at room temperature. The reaction mixture was dialyzed against excess (4 L) 0.1 mol/L NaHCO3 in 0.1 mol/L NaCl, pH 7.6 at 4°C overnight. Approximately 50 to 100 µg aliquot of DTPA modified anti-RAGE $F(ab')_2$ was reacted with 1,296 MBq (30 mCi) of $^{99m}TcO_{-4}$ in 50 µg of $SnCl_2$ in 100 µL of 0.1 N HCl that was flushed with N_2 for 20 minutes. After incubating for 30 minutes, the ^{99m}Tc-anti-RAGE $F(ab')_2$ was separated from free ^{99m}Tc by Sephadex-G25 (10 mL) column (Pharmacia) equilibrated with PBS. Fractions (1.0 mL) were collected, and those fractions containing ^{99m}Tc-anti-RAGE $F(ab')_2$ in the void volume were pooled. The mean specific activity was 48.7±9.3 µCi/µg, and the mean radiochemical purity was 951.6% by instant thin-layer chromatography. The mean injected ^{99m}Tc dose was 3.47 ± 0.29 mCi (128.4 ± 10.73 MBq).

Scan acquisition: SPECT scanning was performed on the nanoSPECT (Mediso, Budapest) running HiSPECT and VivoQuant software (InVicro, Boston MA) using a rabbit bed and replacing the pinhole collimators with 2 low energy ultra-high resolution lead collimators mounted over NaI crystals at 180 degrees. SPECT acquisition parameters: 30 sec/step x 64 steps in 256x256 matrix (1 voxel=1x1mm) in circular 360 degree orbit. Raw data underwent preprocessing using HiSPECT software with medium (35%) smoothing followed by iterative 3.3 reconstruction.

CT scans were performed on Siemens Biograph 64 slice scanner using the following parameters: helical mode, thickness 3 mm, Kv 120, mAs/slice= effective 120, pitch 1.3, collimation 16 x 1.5, rotation time 0.25 sec, matrix 512. The CT scan data was copied onto DICOM files, transferred onto the computer running HiSPECT. The processed SPECT data was merged with the CT data for each rabbit. A fiduciary marker (micro-Curie amounts of ^{99m}Tc in an Eppendorf tube) was affixed to the mid-sternal area of the rabbits to help register the SPECT and CT scans. The CT was used only for anatomical localization of lung boundaries. Because the two scans were acquired on different cameras and at different times, the CT data could not be used for attenuation correcting the SPECT count data.

Image Processing: Filtered and reconstructed SPECT data were merged with the CT data using VivoScope software (InVicro, Boston MA). On the merged images, ROIs were drawn on serial 20 voxel thick transverse slices for the right and left lungs from apex to base. Total counts for each transverse section were recorded and summed for all sections for each lung. The total lung counts were then converted to mCi using camera efficiency, and standard conversion factors. These values were divided by the decay corrected ID to yield %ID for each lung.
For CT image processing, acquisition was performed at 0.4 mm thick slices. Transverse sections were summed into approximately 1 centimeter thick voxel sections. ROIs were drawn around serial sections from apex to base (average 5 sections per lung) and volumes summed in cubic centimeters. Average Hounsfield units for each section were also recorded.

Ex Vivo well counting: Sections from both lungs were dissected and weighed, and the radioactivity determined in a gamma well counter (Wallac Wizard 1470, PerkinElmer, Waltham, MA, USA) and expressed as the percentage of injected dose per gram (%ID/g) of tissue. The radiotracer activity in the samples was corrected for background, decay time, and tissue weight.
